# Making the patient voice heard in a research consortium: experiences from an EU project (IMI-APPROACH)

**DOI:** 10.1186/s40900-021-00267-0

**Published:** 2021-05-10

**Authors:** Jane Taylor, Sjouke Dekker, Diny Jurg, Jon Skandsen, Maureen Grossman, Anne-Karien Marijnissen, Christoph Ladel, Ali Mobasheri, Jon Larkin, Harrie Weinans, Irene Kanter-Schlifke, on behalf of the APPROACH research consortium and APPROACH Principal Investigators, Anne-Karien Marijnissen, Christoph Ladel, Ali Mobasheri, Jon Larkin, Harrie Weinans

**Affiliations:** 1 The APPROACH Patient Council, Utrecht, The Netherlands; 2 Rheumatology & Clinical Immunology, University Medical Center Utrecht, Heidelberglaan 100, Utrecht, 3584 CX The Netherlands; 3 BioBone B.V, Amsterdam, The Netherlands; 4 University of Oulu, Oulu Finland State Research Institute Centre for Innovative Medicine, Vilnius, Lithuania; 5 University Medical Center Utrecht, Utrecht, The Netherlands; 6 GlaxoSmithKline, Collegeville, PA USA; 7 Lygature, Jaarbeursplein 6, Utrecht, 3521 AL The Netherlands

**Keywords:** Osteoarthritis, Patient involvement, Patient engagement, Research consortium, Biomarker, Clinical trial, Public-private partnership, Europe

## Abstract

**Abstract:**

APPROACH is an EU-wide research consortium with the goal to identify different subgroups of knee osteoarthritis to enable future differential diagnosis and treatment. During a 2-year clinical study images, biomarkers and clinical data are collected from people living with knee osteoarthritis and data are analyzed to confirm patterns that can indicate such different subgroups. A Patient Council (PC) has been set up at project initiation and consists of five people from Norway, The Netherlands and UK. Initially, this group of individuals had to learn how to effectively work with each other and with the researchers. Today, the PC is a strong team that is fully integrated in the consortium and acknowledged by researchers as an important sounding board.

The article describes this journey looking at formal processes of involvement – organizational structure, budget, meetings – and more informal processes such as building relationships and changing researcher perceptions. It describes how the PC helped improve the experience and engagement of study participants by providing input to the clinical protocol and ensuring effective communication (e.g. through direct interactions with participants and newsletters). Furthermore, the PC is helping with dissemination of results and project advocacy, and overall provides the patient perspective to researchers. Additionally, the authors experienced and describe the intangible benefits such as a shift in researcher attitudes and a sense of community and purpose for PC members. Importantly, learnings reported in this article also include the challenges, such as effective integration of the PC with researchers’ work in the early phase of the project.

**Trial registration:**

US National Library of Medicine, NCT03883568, retrospectively registered 21 March 2019.

**Supplementary Information:**

The online version contains supplementary material available at 10.1186/s40900-021-00267-0.

## Plain English summary

This article describes the activities and lessons learned from the involvement of a Patient Council in APPROACH, a 5-year European clinical research project focusing on osteoarthritis, the most common form of joint disease. The Patient Council is a group of five people from different EU countries who live with osteoarthritis. They use their knowledge of life with the disease and their own past experience as participants in clinical studies to help improve the experience of people who participate in the APPROACH clinical study. In addition, they provide the overall patient perspective to the researchers within the project.

When the project started, the Patient Council was a group of individuals who didn’t know each other. They had to find a way to work together with each other as a team, and with the researchers to ensure their involvement was integrated effectively into the project. The authors (current members of the Patient Council and other selected project members) describe in this article what was needed to successfully work together, the process of becoming fully engaged and involved and describe the impact that their activities have made on the clinical study during the project and beyond. They share their lessons learned with the goal to help other research projects to integrate the patient perspective effectively, and to encourage people living with a medical condition to share their experience with researchers through patient involvement activities.

**Supplementary Information:**

The online version contains supplementary material available at 10.1186/s40900-021-00267-0.

## Introduction

In the specific area of arthritis and other musculoskeletal diseases patient involvement has been shown to contribute to more relevant patient outcomes in clinical trials as well as changing the scope of rheumatology research [1–3]. In this paper we discuss both the experience of patient involvement in the APPROACH clinical study - and the impact it has made on study design and participant experience - and on the wider aspects of the project. The learnings are presented as a case study for improving and refining patient involvement in other international clinical studies across many disease areas.

The Guidance for Reporting Involvement of Patients and the Public (GRIPP 2) Short Form [4] is included as Supplementary file 1.

### The APPROACH project

The Applied Public-Private Research enabling Osteo Arthritis Clinical Headway (APPROACH)^1^ is an international and cross-discipline collaboration project bringing together clinicians and researchers from different disciplines (rheumatology, orthopedics, imaging departments and laboratories), from clinical centers, basic research institutes and pharmaceutical and non-pharmaceutical companies, as well as people diagnosed with osteoarthritis (OA). The project has been running from June 2015 to December 2021 and is funded by the Innovative Medicines Initiative (IMI), a partnership between the European Commission (EC) and the European Federation of Pharmaceutical Industries and Associations (EFPIA) that funds health research and innovation [5].

OA is the most common form of arthritis and its incidence increases with age; by 65 years approximately 80% of the population has some radiographic evidence of OA [6, 7]. OA is characterised by joint pain and gradual loss of function, and significantly impacts the quality of life of patients and their families [8]. Despite the impact of the disease on individuals and healthcare systems, there are currently no disease-modifying OA-specific treatments authorized for clinical use [9]. Unfortunately, many clinical trials fail [10] and almost every OA clinical trial conducted to date has failed to produce conclusive results, which means that the drugs and interventions being tested are unlikely to be approved by regulatory authorities. Evidence supports the hypothesis that outcomes of OA trials are frequently inconclusive because patients enrolled are too heterogeneous [10]. In line with this, the primary objective of APPROACH is to improve future OA trial design by collecting data that in due course can lead to more specific selection criteria for trial inclusion or the use of more specific and sensitive outcome parameters defining progression of OA.

**Fig. 1:**
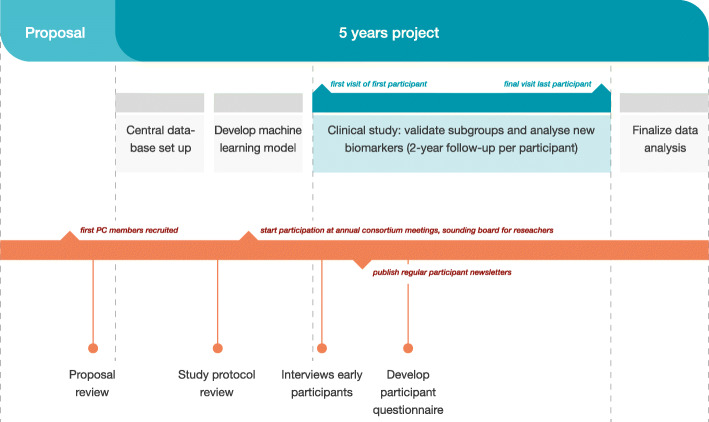
Main project activities (blue) and Patient Council activities (orange)

In order to achieve its goals, a 2-year clinical study has been set up by the APPROACH consortium in France, The Netherlands, Norway and Spain [11]. From March 2018 until April 2019 a total of 297 participants have been included (last visit of the last participant expected in April 2021). The participant inclusion procedure was based on unique machine learning models to increase the likelihood of radiographic joint space width loss and/or knee pain progression during a limited, 2-year follow-up period [11]. Parameters measured at baseline, 6, 12 and 24 months include clinical and imaging parameters, questionnaires and biological markers in blood and urine [11]. See Fig. 1 for a high level overview of project and Patient Council (PC) activities.

### The European dimension

**Fig. 2:**
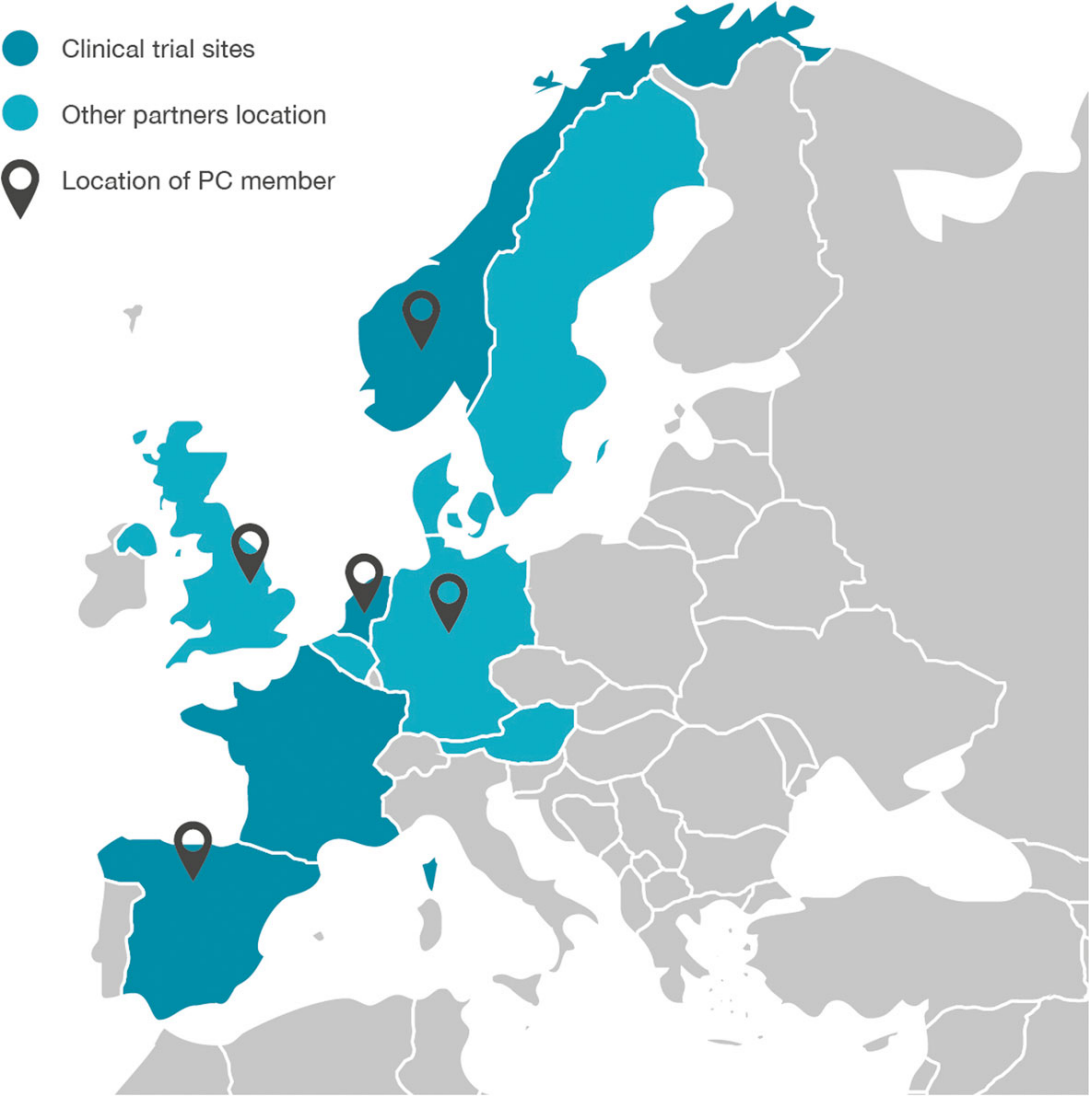
Geographic distribution of consortium partners, clinical sites and (former and current) PC members

APPROACH is a truly European collaboration, not only in terms of funding but also considering the research partners involved. The clinical study is executed in France, The Netherlands, Norway, and Spain, and further research partners involved are based in Austria, Belgium, Denmark, Finland, Germany, Sweden and the United Kingdom (see Fig. 2).

Such broad international collaboration is of great benefit to both the project itself as well as for future implementation of findings. It brings together the most suitable expertise from across Europe, and at the same time challenges the team to consider the different clinical settings, health systems and cultures. From the beginning it has been important to the patient experts that the Patient Council (PC) they formed should reflect this international variety.

## Setting up patient involvement in the project

The issues in OA drug development are large and complex. One way of addressing these challenges is the model of a public-private partnership of engaged, knowledgeable and complementary industrial, academic and patient partners. In addition, there is extensive published evidence to suggest that involving people with lived experience of a condition in all stages of clinical trial design, both for interventional and observational studies, is important for successful implementation, outcomes and future decision-making [12–16]. This is also recognized by both EFPIA and IMI who have made active efforts to increase the involvement of people living with the condition studied in research projects they fund [17–19].

In line with this, the importance of having a strong patient voice in APPROACH was recognized from the start. At the project’s inception, a PC was established to ensure patient involvement throughout the duration of the project. Following the INVOLVE guidelines, the authors define patient involvement as active involvement of patients in the research project and with research organizations. This is in contrast to patient participation (where people take part in a research study) or patient engagement (where information and knowledge about research is provided and disseminated) [20].

*‘There was clearly a commitment to patient involvement from the key research leads from the outset of the study but I think there was less knowledge of what good patient involvement looks like.’ (PC member, UK)*^2^

While the support for a PC was evident, establishing such a group in practice and fully integrating it with the activities across consortium members required proactive effort.

### Patient involvement at the proposal stage

Two organizations representing patient interests were involved as consortium partners from the beginning, and as such in shaping the proposal: *ReumaNederland* in the Netherlands (previously called Reumafonds) and *Versus* *Arthritis* in the UK (previously called Arthritis Research UK).

Once the proposal was close to submission, individual patients were invited through these two organizations to review the proposal and provide input. This initial group consisted of 5 people diagnosed with OA: two people from the UK who had previously been involved in shaping and reviewing research, two people from the Netherlands who had experience as participants in another longitudinal clinical study called ‘CHECK study’ [21], and one person from Germany who had been actively involved in the EULAR Standing Committee of PARE, a group of representatives of rheumatic and musculoskeletal user groups from across Europe.

### Establishment of the PC after project launch

Once the project was officially launched, the members from UK and The Netherlands remained involved to form the initial PC. This meant that the PC was at first structured based on the location of patient organizations involved as project partners. However, considering that the bulk of the PC activities was expected to center around the clinical study, the PC members felt it was important that more countries where clinical sites were set up were represented in the PC (Spain, Norway and France, in addition to The Netherlands). An active effort was made to reach out to patient groups in those countries. In the course of the first 3 years of the project, a member from Spain and a member from Norway joined the PC. Attempts were made to also add a member from France, but these were not successful.

In addition, if and where possible, the PC team aimed at putting together a diverse group of people, with different clinical manifestations of OA, broad age distribution (20–75) and various occupational and life experiences.

### Operationalizing the PC

Initially, the PC was a group of individuals who didn’t know each other and that had no opportunity to interact face to face prior to starting the work on the project. The scope of their activities was not clearly defined, and their status within the project not yet anchored. PC members considered this a limitation to contributing to the project effectively. This was an important reason for the PC to request their contact person, the PC coordinator, to develop ‘Terms of Reference’ (see Supplementary file 2). These Terms of Reference (developed in 2017) provided an initial framework for the role of the PC in the project, and established its relationship with other consortium partners. At the same time budget was reserved for compensation of PC members’ time and their travel costs to annual consortium meetings. As a result of this, the PC started to have more frequent interaction as a group, and to get to know each other and the members of the consortium better. Having more opportunity to share ideas in a formal way, the PC gained confidence in their own role as well as in the support of the consortium. At the same time, PC members started to get more insight into the research aspects of the project, and to see opportunities where their input would be helpful. This resulted in the PC taking on a more pro-active role; they asked for example to be involved in the annual meetings, in editing the informed consent, editing the participant newsletter and in dissemination. At annual meetings, the PC gave presentations of their experience of living with OA and the work of the PC to the entire consortium in the same way as the leaders of the research work packages. Their prominence at those meetings helped emphasize the support the PC was increasingly getting from the consortium leadership. Such acknowledgement of the importance of the PC also encouraged the continued and open dialogue with all consortium members, at annual meetings and beyond.

As the PC evolved, different roles surfaced within the group: The Communicator, The Facilitator, The Critic, The Caretaker (of study participants) [22]. Recognizing and making use of the different skills and experiences of PC members strengthened the group, their sense of team spirit and confidence in the role they could play within the consortium. The group started to take more ownership of their scope and to pro-actively take initiative. Locally, PC members also strengthened their relationship with respective clinical study teams. In keeping with the research on effective group working, the members found that maintaining the group size at around 6 was effective in enabling participation and decision making [23].

Today, 5 years into the project, there is uniform appreciation in the consortium for the important contributions of the PC, as reported for example at the 2019 annual meeting (see Suppl. File 5).

As Jon from the PC said, *‘Scientists go by evidence – so it will take time to produce the evidence and convince people of the value of the PC. But in APPROACH we have shown that this can happen, and that as patients we have the power to drive this change.’*

PC input in project deliverables such as the clinical study protocol or communication has been very tangible (see Study design and Participant engagement and retention sections). However, their less tangible contribution to the project may be even more important: direct interactions with the research partners have resulted in a different mindset of project members. The balanced relationship that has consecutively developed between the PC and research partners has been of great value and a big achievement for the entire consortium.

*‘We scientists think in numbers’,* one researcher explained at the annual consortium meeting in 2019, *‘you [the PC] are challenging us and keeping us real.’*

## PC activities: improving science and participant retention

### Underlying perceptions & language

OA affects more than 40 million people across Europe and is the fastest growing cause of disability worldwide with more than 300 million cases of hip and knee osteoarthritis reported globally in 2017 [24]. Yet, it is often misunderstood and seen as an inevitable part of the ageing process, and the effects on individuals and society are not appropriately recognized. One of the key tasks of patients involved in a project like APPROACH is to help researchers understand what the reality of life with a condition like OA looks like. In line with this, patients should alert researchers to unhelpful perceptions, practices or use of language.

*‘We gave members of the project the opportunity to meet people who actually lived with OA and enabled them to understand more of the impact of OA on our lives. Maybe we gave them an added incentive to get on with it.’ (PC member, UK)*

The initial PC group reviewed the project proposal before submission to the funding agency. At that point their feedback was mainly focused on the language used. For example, the term ‘participant burden’ was perceived as negative by the patient experts. Researchers used this term to describe what happens to study participants, but from the perspective of the patients it positioned participants as ‘passive sufferers’, which they didn’t feel was appropriate. The language in the document was adapted and the terms ‘difficulties of OA’ and ‘barriers to participation’ were introduced to avoid ‘participant burden’.

This early input of patients triggered an important conversation about use of language, which is a recurrent and fundamental topic in patient involvement [25, 26]. To exemplify this, Jane, a PC member from the UK, shared her personal experience on the power of language:

*‘I was told more than once by doctors that I had ‘failed’ on a particular drug adding to my sense of inadequacy about having a long term illness in the first place. The reality was that the drugs had ‘failed’ me’.*

The way language reinforces existing power relationships was addressed by the PC throughout the project and in an early presentation to researchers. Highlighting this topic made an impact on the terminology used by the consortium members.

*‘I stopped using the word ‘subject’ and used ‘participant’ instead. Originally I was told off, subject is not necessarily a participant yet, only a candidate so in a strict meaning more correct. But it sounds awful.’ (Researcher, France)*

Throughout the project lifecycle, the PC continued to raise awareness of the reality through describing their lived experience, including presentations on the current limitations of measurements of pain. Researchers were able to put real and diverse people in place of disease types.

*‘Harrie, one of the Principal Investigators, used to present a PowerPoint slide showing 3 individual OA knee scans to illustrate the variation between OA patients. A few years later, in a joint presentation I did with him he put up this slide as a way of stating how he used to see patients. He then put up a photo of the PC as an example of how he now thinks of people living with OA’ (PC member, UK), see*

**Fig. 3:**
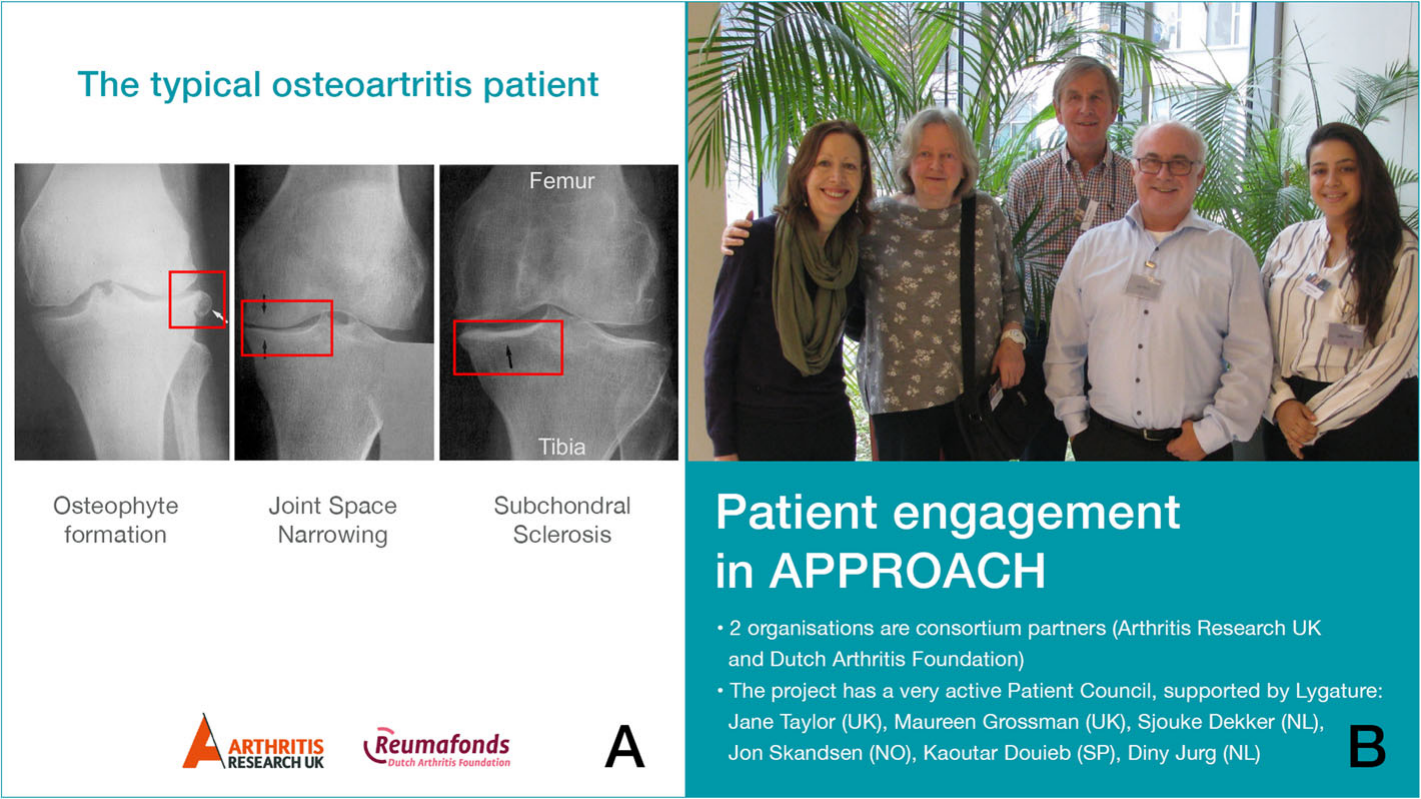
Two ways to see a patient. **a** OA knee radiographs to illustrate the variation between OA patients. **b** Group photograph of the APPROACH PC (2018) to illustrate that patients are more than knee radiographs

Fig. 3*.*

There are crucial differences in how researchers, clinicians and patients frame and understand a disease. Researchers have looked at how patient narratives can challenge conventional wisdom and generate new hypotheses [27, 28]. Patients sharing their experience of living with an illness, as the PC has done in APPROACH, can also challenge the privileging of the biomedical model and create a more pluralistic way of knowing - an important shift in moving towards genuine co-production in research [29].

*‘With new members of the lab, I now discuss not only the science of OA but also how it affects the patient.’ (Principal Investigator, The Netherlands)*

### Study design

#### Review of the study protocol by PC members

APPROACH has a substantial clinical study component to it. When reviewing the clinical protocol, PC members looked at the practical experience of participants when they would visit the hospital (site visits) and also considered integral issues such as incidental findings.

The PC gave their input to researchers on the ethical and practical concerns around interpretation of radiographs and what to do if possible findings indicated medical problems. Eventually, a good process was developed jointly between the researchers and the PC, whereby APPROACH clinical researchers can hand out MRI images and radiographs but will always refer participants to their treating physician for further discussion.

The fasting blood sample collection was another example where feedback from the PC resulted in a change. According to the original set-up, participants needed to fast prior to blood samples being collected at the initial visit. However, in centres where participants had significant travel time to the clinic this created potential issues for the participants that seemed unreasonable to the PC members. The PC therefore proposed to arrange for these participants to have someone come to their home and draw the blood early in the morning so that they didn’t need to fast for too long. At first, costs of this approach and inconsistency of blood collection timings were a concern to the consortium. However, ultimately the PC was able to argue for the importance of this change for participants, and their suggestion was implemented.

#### Dry run of clinical protocol by APPROACH researchers

Three research members of the APPROACH consortium participated in the MRI test and re-test study in four of the clinical centres in the Netherlands, Norway and Spain. Effectively this was a dry-run through the MRI protocols and as such provided an opportunity to experience the protocol that study participants would follow. Having conducted this dry-run in July, the researchers experienced temperatures of up to 36 °C, giving them an understanding of the unpredictable factors that can make the protocol more arduous for participants. While this was an activity initiated and executed by researchers (two of them co-authors of this paper) rather than PC members, it turned out to be an important tool to increase awareness of participant experience amongst the consortium members. However, actual changes made due to this dry-run were focused on technical considerations and not aimed at improving the participant experience. For future studies, the authors recommend that a similar dry-run of the protocol would be conducted, ideally by researchers and PC members together, followed by a collective discussion on possible changes needed to the protocol to also improve the participant experience.

#### Interviews with early study participants

The PC and researchers felt it was important to have a solid understanding of the experience of the first study participants early, so that changes could be made to improve the experience for the remainder of the study. Therefore PC members in Norway and the Netherlands arranged and conducted personal interviews with study participants, who consented to this upfront and with feedback collected anonymously. Their feedback led to more changes to the logistics of initial and subsequent visits in terms of time of day, weekdays versus weekend, and provision of refreshments.

It is important to note that changes to the actual study protocol weren’t possible at this stage, as amendments to clinical protocols have to undergo review by ethics committees, which is a time consuming process. However, some circumstances can be adapted in the course of an ongoing clinical study to improve e.g. waiting time, waiting room conditions, schedule of visits (not too late), and information provided. (e.g. timespan, catering, parking). This was where the PC provided most suggestions for improvements based on feedback of early study participants.

*‘With the feedback of study participants we learnt some details on the clinical site visits that we hadn’t considered previously. We have tried to now improve things in the organization of the visits’ (Clinical Researcher, The Netherlands)*

Published evidence from a systematic review suggests that implementation of strategies that aim to reduce so-called ‘participant burden’ may be effective in maximising cohort retention [30]. From 279 participants included in the APPROACH study initially, 263 are still enrolled in the study (some participants did not want to come to the hospital for their recent visit due to COVID-19) out of which 112 have already completed their participation in the study.

#### Ongoing support to local clinical sites

As relationships between PC members and local clinical sites developed, researchers connected to the PC more often ad hoc to receive input on topics that have come up. For example, when starting up visits in Norway after the first wave of COVID-19, the Norwegian PC member was asked to provide input on the proposed plan for carrying out the visits with new precautions in place. Many people living with OA belong to a vulnerable group, e.g. due to their treatment regime or medical history. The PC reviewed the plans with that in mind and could provide a number of suggestions to researchers based on practical and emotional needs of patients during the COVID-19 crisis. Specifically, the PC advised to reduce the number of participants received per day, to check with participants upfront if they were able to travel to the site safely and to take extra precautions to clean materials and devices used.

This contribution was an example of how the PC can respond to unexpected changes, and how important a good collaboration between researchers and patients is in order to make use of their input.

### Participant engagement and retention

#### Participant questionnaire

One year after the start of the study, researchers asked the PC to help develop an evaluation form for participants, with the aim to evaluate and further improve their experience. For this, a Dutch PC member interviewed two local participants at the study site. Interviews took place face to face, and all feedback was anonymous. Based on these interviews the PC, in collaboration with researchers, developed an evaluation form for study participants (see Supplementary file 3) including questions on practicalities at the study site (e.g. if research department was easily accessible), communication before/after visits, and overall experience of visits.

A total of 181 participants from four study sites in France, Spain and The Netherlands anonymously completed the evaluation form. Over 90% of the respondents were satisfied with the study set-up and logistics. The most important items for improvement were reduction in the number of visits and imaging time. Participants also asked if it was possible to give more information on their individual health status. Based on participant feedback, the research-physicians provided more information in the invitation letters to better prepare people, such as the timespan of the visit and the availability of catering.

The PC also suggests another evaluation at the end of the project to learn for future projects.

#### Participant newsletter

In order to keep study participants informed and engaged, a ‘participant newsletter’ was established in the first year of the clinical study. This newsletter is published twice a year, translated from English into the languages of countries where clinical sites are located, and hard copies are sent to those sites for distribution.

The content of these newsletters gives insight into the people behind the project, information about relevant topics such as the techniques used in the study, and updates on the study progress. The goal is to make the information both interesting and easily readable, so that it encourages participants to remain involved with the study.

*‘Participants are enthusiastic about the newsletter, they think it is very interesting to learn more about the research they are involved in.’ (Clinical Researcher, The Netherlands)*

The newsletter editorial team consists of one member of the PC that is permanently on the team, another 1–2 PC members that rotate, three members of the research team and the communication staff member from the programme management organisation who leads the effort. The PC members have been involved throughout in writing and reviewing content and design layouts, and reviewing translations where possible. This involvement of the PC has been of great importance to ensure that the newsletter is ‘patient-centric’ and gives a sense of the APPROACH community where participants, researchers and the PC all work together to improve OA care in the future.

An example of a newsletter can be found in the supplementary materials (Supplementary file 4) or at the APPROACH website.

*‘The PC can facilitate good communication like we have done for example with the newsletter.’ (PC member, Norway)*

It is important that communication with participants doesn’t stop after their input into the protocol is finished. Most participants want to get feedback on the study they contributed to and to learn about the results. This is important in recognizing and respecting participant input throughout the study and also encourages people to take part in future clinical studies. Despite guidance at both national and international level of the ethical imperative to feedback results to participants [31], studies have shown that many participants never get feedback [32]. Furthermore, *how* to communicate results is as important as *what* to communicate and it is often not something considered by researchers [33]. This is an area where patient insight can be very important, as APPROACH consortium members also highlighted in their feedback to the PC at the 2019 annual meeting, and it is something that the PC will be taking forward in APPROACH.

‘*Help us design easy-to-read communication towards the participants. It would be a shame to produce a report and have the participants discard it because it is unintelligible.’ (Researcher, The Netherlands)*

#### Peer support for study participants

The PC members also suggested facilitating interaction between study participants where possible, and to be available for participants to connect to the PC directly. From their own experience PC members knew that participating in a clinical study can be a lonely task, and that connection with peers can help reduce the sense of isolation.

Therefore, in addition to keeping people informed via the newsletter, participants are encouraged to reach out to the PC in case they would like to have peer support, or have any questions regarding PC activities in the project. For this, a dedicated email inbox has been set up that is managed by the PC coordinator.

*‘The Patient Council being willing to be a point of contact for concerns was another means of “looking after” the study participants, the willing subjects of so many procedures.’ (PC member, UK)*

To date few study participants have made use of this option in APPROACH. For future studies, the authors recommend exploring different ways to connect with participants to ensure their needs are understood and met.

## Lessons learned

When APPROACH was launched in November 2015, the PC was a group of individuals who had to learn how to work as a team and what role they could play for the project. Over time, this has changed and the PC has become an integral part of the consortium.

*‘What we have learned is that setting up a functional PC does not happen overnight. It costs time and effort, and it needs proper preparation and coordination. But it is all worth it!’ (PC coordinator, The Netherlands)*

**Fig. 4:**
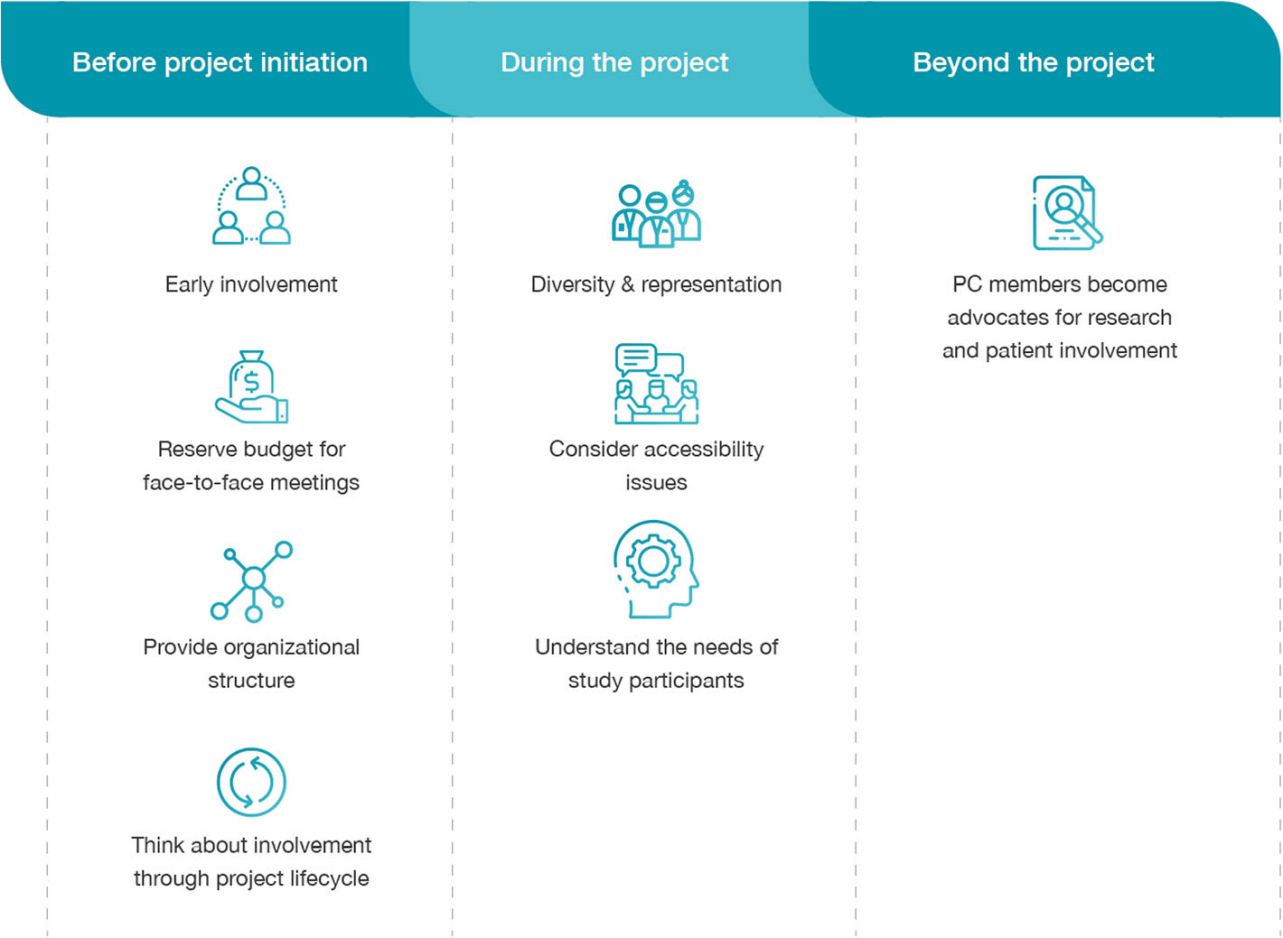
Summary of lessons learned and recommendations for future projects

In this section, the authors share what they have learned throughout that process, describing benefits but also limitations of their own approach. They provide quotes and descriptive statements (rather than numbers or formal guidelines) and important lessons that can help others set up patient involvement efficiently and effectively from the start (Fig. 4).

### Before project initiation

#### Involvement

Whatever the nature of a project, it is essential that the patient voice is represented in early discussions when formulating the concept and idea. Evaluation of the impact of early involvement on the research process suggests a range of benefits such as better identification of relevant questions, more credibility of the knowledge produced and better application of results to specific contexts [20, 34–37]. Benefits of involving patients to review the proposal are described under Underlying perceptions & language. However, even in case of APPROACH patients could have contributed more had they been involved sooner, i.e. during initial discussions shaping the bid.

In practice, it can be challenging to find individual patients who can contribute at this early stage of a project. Patient organizations at European or local level are a good place to start, as was done in APPROACH. However, as described under Establishment of the PC after project launch, the initial PC that was formed using this approach didn’t entirely represent what was needed for the project later on (i.e. representation from countries where clinical studies took place). To address this researchers had to reach out to local groups, hospitals and community organizations or contacts within their institutions who have some responsibility for public engagement.

#### Reserve budget for face-to-face meetings

A common issue of patient involvement is lack of appropriate funding [38, 39]. It has been established as good practice to offer patient advisors an honorarium payment of some kind and there is advice on this, for example, from INVOLVE [20]. In addition, budget for travel and other expenses needs to be included from the beginning in order to facilitate in-person meetings. This is especially important at early stages of a project.

*‘Meeting the PC face to face has meant I interact directly with patients, something not done before. This has helped me understand real needs, limitations and ‘humanize’ biomedical research.’ (Researcher, UK)*

In case of APPROACH, travel of the PC to annual meetings was not initially accounted for, both in terms of budget and meeting planning.

*‘For next time I would suggest: at first meeting each other in real, so you can get to know each other a little bit before having meetings via phone or video.’ (PC member, The Netherlands)*

Once relationships are established, it is easier for the PC to function as a team remotely. Research shows the importance of some element of ‘social presence’, the degree to which an approach to communication helps people feel a personal connection with others [40]. The more social presence that exists in team communications in general, the more the personal relationships between team members can develop. Although virtual meetings can develop this, a face to face meeting has a high level of social presence due to normal conversational interaction.

*‘These face to face meetings are so important, particularly if you don’t share the same language. It is the informal chats with researchers that make the meetings easier and create feeling of all working as one group.’ (PC member, Norway)*

#### Provide an organizational structure

Individual patients that form the PC are not connected through a structured, professional organisation with its resources and infrastructures. Solid management support for the PC is essential for it to become integrated in the consortium and operate effectively. The assigned PC coordinator should take the lead in setting up meetings and ensuring that infrastructures such as video calling technology are accessible to PC members. In addition, the PC coordinator should, at the start of the project, mediate discussions to align expectations from all parties involved. This way PC responsibilities should be clear and realistic, and ultimately be formalized as Terms of Reference. The authors also recommend revising and, if applicable, updating Terms of Reference on a regular basis to ensure that it continues to be a valid reference document as the project develops [41].

*‘It seems to me that it took some time before the project management, the project members and the Patient Council actually found the tasks, responsibilities and working form for the PC. I think everybody would have benefitted if the work had been clearly defined earlier.’ (PC member, Norway)*

The authors also strongly recommend establishing regular communication between the PC and the project management from the beginning. It’s important to maintain contact with everyone even if it is just to say nothing much is required from them at the moment. If that doesn’t happen the involved patients may become detached from the project.

The APPROACH PC initially was struggling with this, particularly when there were changes in the management. When Terms of Reference and regular communication were established, and when the group organised itself and appointed a Chair, this made for much greater cohesion of the group and sense of empowerment and motivation [41]. While the method of working needs to be established early on, it should also be evaluated and adjusted along the way if needed. As the project evolves, the expectations of both PC members and other project partners may change and it is important to keep the conversation about this open.

#### Think about involvement through the project lifecycle

The shorter term activities are easy to define upfront but it is more challenging to think about sustained involvement across the entire project. However, people with ‘real-world’ experience of living with a condition can provide a very different perspective to that of researchers at all stages of the project – from shaping the proposal to disseminating and publicizing the results, shaping any follow-up projects and contributing to longer-term advocacy activities. In the case of APPROACH, PC activities were initially focused on study design and participant engagement. As the project evolved, and when PC input on these items became less important, the PC pro-actively re-defined in what way they could contribute at the next stages of the project. This led to the PC session at the 2019 annual meeting (Supplementary file 5), where input was gathered from all consortium members.

*‘The PC could help us with thinking about the future use of the study results. If we want to validate the outcomes from a clinical study, then we need advice on how to design a new study and how this tool can help doctors and patients in the future. This will help in shaping something that is actually effective in the clinic.’ (Researcher, Denmark)*

For future projects, defined milestones and deliverables can be taken as a useful framework for planning patient involvement in a meaningful and impactful way at key stages, considering the entire project lifecycle. Having made appropriate plans upfront for patient involvement also makes it easier to ask for budget accordingly, which in turn is essential to conduct patient involvement successfully.

### After project kick-off

*‘The humanity, compassion and unique perspective the PC brought to the project was crystal clear from the kick-off onwards. Their contributions had both a disruptive and stabilizing effect. Through challenging scientists/clinicians to think differently about the research being conducted, they provided a patient-focused vision for others to follow.’ (Principal Investigator, USA)*

#### Diversity and representation

The term ‘diversity’ covers many aspects including gender, age, ethnicity, cultural, educational and socio-economic background, and clinical presentation. Ideally, the composition of the PC takes into account all those aspects. In practice, this is often not feasible. The project members have to define together what the most important aspects are to consider, and what level of diversity is essential, and what is possible.

This should also take into account experiences relevant to the role the PC is expected to fulfill in the project. In APPROACH, PC members from the UK had prior experience as patient advocates, whereas members from the Netherlands had previously been clinical study participants themselves. Having both aspects represented in the PC was an important asset for the group.

It’s important to remember that diversity should not be a goal in itself and needs to be balanced with representation. In context of APPROACH, ‘representation’ means including PC members from countries where the clinical study was conducted. APPROACH is an international project and priority was given to representation from the different countries, and to establishing a cohesive group of 5–7 people. The authors recommend that where possible, international projects take into account the most striking cultural differences across the region, such as ‘southern’ and ‘northern’ cultures and seek to have appropriate representation of those in the PC.

Furthermore, in APPROACH, where English is the common language, a certain educational level was a pre-requisite which limited diversity of social backgrounds. Ideally, one would establish ways of gathering input from a more diverse group of patients while maintaining the representation of different countries, for example by organizing local focus groups and bringing that input back into an international PC.

#### Consider accessibility issues

Striving for diversity and representation goes hand-in-hand with making an active effort to ensure accessibility. While physical accessibility is obviously important, there can be other barriers too. For example face to face meetings should be economically accessible and expenses should be paid upfront or arranged directly with hotels or airlines [41].

A practical point that is often overlooked is the length and timing of meetings. The meeting organizers should check upfront what patient requirements are so that for example start time, breaks and flexibility for patients to take a break if need be are arranged accordingly. Especially in relation to musculoskeletal health it is essential to consider the importance of breaks and physical activity and the chance to walk around, stretch the muscles and lubricate the joints.

It is also important that people feel the set-up of their involvement is psychologically accessible. Ensure there are enough patient representatives to support each other, so that one individual doesn’t feel psychologically isolated.

Linguistic barriers worked in two ways in APPROACH. Firstly there is a lot of complex science in the project. It is all too easy for researchers summarizing their work to talk only to their peers. Before the third annual meeting the PC requested that each of the speakers produced a basic plain English summary and glossary of terms for everyone [41]. This made a real difference in PC members being able to contribute more actively and often critically comment and question the researchers. In fact, this had a positive effect also on all researchers from various disciplines in creating more inclusive discussions and interaction.

Each PC member needs to get their voice heard and feel comfortable with their role in the group. Dominance of English as default language inevitably has an effect on this. UK members of the PC experienced a tendency to dominate the conversations, particularly when meetings were held by phone.

*‘I know now that I did not make allowances for the fact that I was working in my first language and others were not, but we live and learn.’ (PC member, UK)*

*‘Working with people from other countries enriched on the one hand; on the other hand it made the communication more difficult because in the discussions a lot of nuances disappeared.’ (PC member, The Netherlands)*

A key learning point was to put as much information as possible in writing in advance of meetings, to give people time to think about responses upfront, and to collect written feedback on follow up items afterwards, to ensure that every PC member has the opportunity to provide their input. At annual meetings there was a tendency for the UK speakers to initially present the work of the PC because it seemed easier for them than those for whom English is not their first language. At the 3^rd^ annual meeting the PC devised a question and answer interview format between two members of the PC from Norway and the Netherlands. This made it easier for them to talk about their work that year and the audience enjoyed the different format.

#### Understand the needs of study participants

If the project involves a clinical study, like APPROACH does, it is important to interact with study participants. Firstly, so that the PC can learn from them, represent their voice in the consortium and help make their experience as comfortable as possible. Secondly to keep participants engaged in the project and motivated to continue following the study regimen.

In APPROACH the PC tried connecting with study participants in various ways: through the newsletter, one-on-one interviews at the clinical sites and by inviting them to connect through a dedicated email address. However, the PC found it difficult to make direct contact, the email inbox was barely used and there was no direct feedback on participant newsletters.

*‘Unfortunately, the research hospitals were all in the Western part of the Netherlands, relatively far away from my home, so to me the distance to study participants felt considerable. I would have liked to be more of a direct supporter for them.‘(PC member, the Netherlands)*

In APPROACH, the Participant questionnaire and Interviews with early study participants proved to be the most effective way of connecting with study participants and ensuring their voice was heard. In addition, the Dry-run of the clinical protocol by APPROACH researchers was an excellent way to gain insight in the participant experience and is recommended to conduct wherever possible.

*‘We ask people to do a lot without experiencing it ourselves. When we tested the protocol we understood what it meant to spend 1 h in an MRI. ‘(Researcher, Germany)*

In hindsight, the authors believe that focus group meetings could have been organised at clinical sites to broaden the group of participants providing feedback to the PC. This may be an option for future clinical studies.

#### PC members become advocates for research and patient involvement

**Fig. 5:**
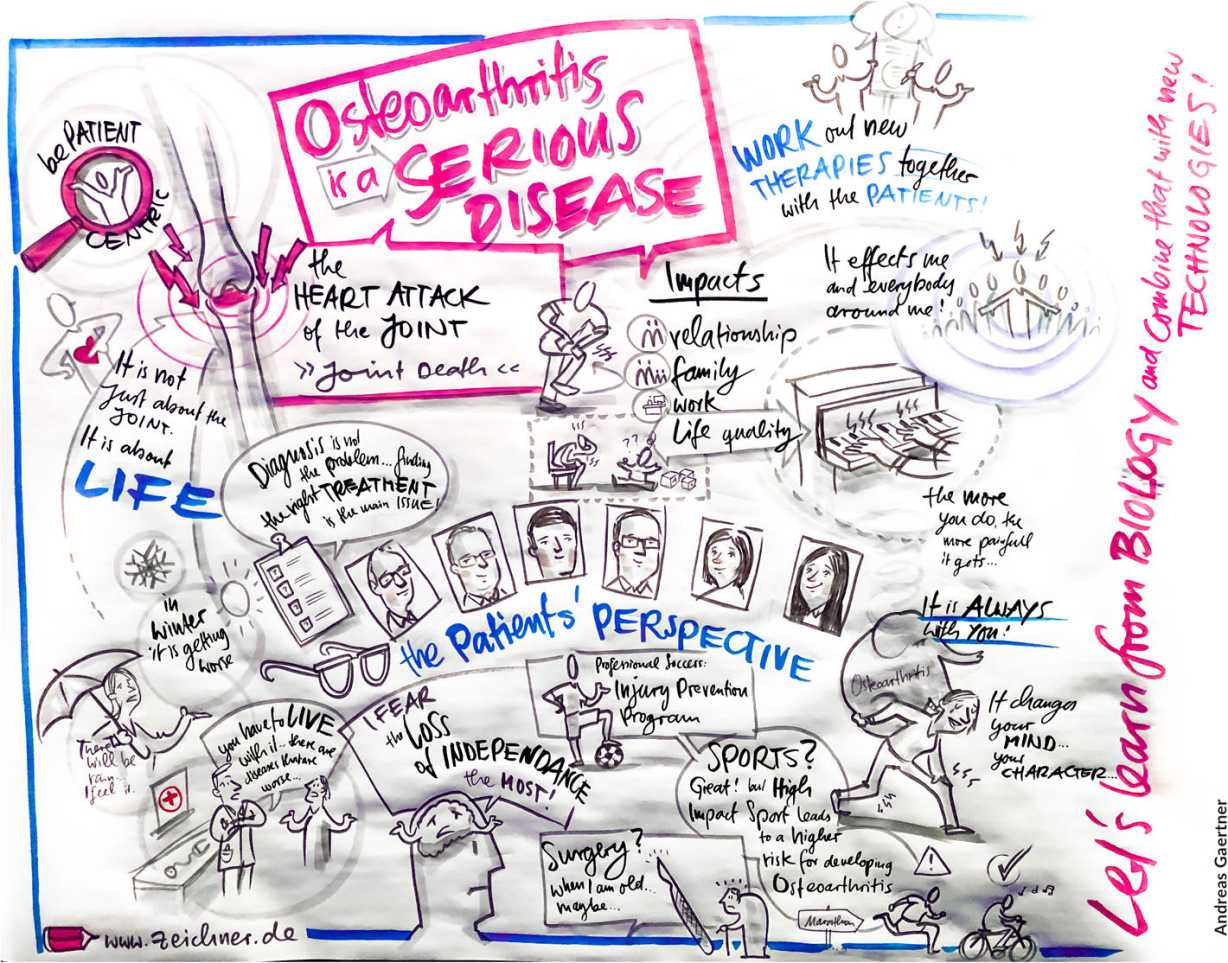
Professionally drawn doodle schematic from Merck 350th anniversary research day illustrating the patient perspective, as provided by APPROACH PC members that day

Importantly, PC members realized that they have also become advocates beyond the APPROACH project. They continuously work on raising awareness in various fora, and advocate for the importance of research in OA and of patient involvement as an essential part of such research activities. PC members gave, for example, presentations to wider audiences (beyond APPROACH consortium members) in the UK, The Netherlands, Spain and Norway, as well as on European level at a symposium in Brussels and at the headquarters of Merck on the occasion of their 350th anniversary research day (see Fig. 5). One member addressed parliament in the UK as part of an All Party Parliamentary Group on life sciences, stressing the importance for patients of continued UK funding for international research.

At that meeting, the PC used the APPROACH study and its own activities within the consortium as a vehicle to increase awareness of OA and of patient involvement. Interestingly, in feedback from researchers this was one of the areas they thought of as important for the PC, as one researcher from Germany emphasized: *‘Continue disseminating the project work to a wider audience, both at European level and in individual countries. Explain to politicians and people who live with OA why work like this is critical for the future of personalized healthcare.’*

For future projects the authors advice that PC members take up this advocacy role actively and consciously.

## Conclusions

While the impact of PC activities has not formally been measured in APPROACH, feedback from researchers and positive participant questionnaire responses indicate that the PC has made a noticeable contribution. See supplementary material 5 for description of the activity with consortium members at the 2019 annual meeting where feedback on PC impact was collected in a focus group setting.

In addition, there may be indirect benefits of patient involvement, such as putting the need for OA research on the political agenda. These indirect benefits may not often be measured or evaluated but may be as important and even further reaching than direct benefits.

The presence of patients also changes the way researchers talk to each other - having to think about and talk about their research in lay terms encourages more self-reflection on “what”, “why” and “how” of what they are doing. This also encourages more discussion across areas and subject disciplines from other researchers in the room. This can lead to a subtle change in the dynamics in the room and make all discussions more inclusive.

*‘The questions asked by the patient council are sometimes very surprising, something that researchers have not thought of before. It opens new roads for the design of new studies.’ (Researcher, UK)*

It is important for researchers to get used to having people with lived experience in the room contributing to research. This is true at all levels but especially for younger people starting out in research. Getting to know the people behind the data can change the perspective of researchers [37, 42].

*‘As the project progressed, I noticed that PC members were being included and consulted in research conversations in areas in which they were not formally involved. It seemed as if researchers got used to us being around.’ (PC member, UK)*

Patients, in this case PC members, also personally benefit from their involvement by having their experience recognized and validated [37]. Having a chronic disease can often be disempowering - a series of having to give things up. Being able to use your experience to enhance the value of research can give back a sense of control and a stake in your own future.

*‘Absolutely positive! ‘It has been a learning experience for me as an individual, and participating in such a large and international project has given me a bit more self-confidence.’ (PC member, Norway)*

*‘I was not looking forward to being part of the editorial team producing the newsletter, but I actually found I had ideas and enjoyed myself. I had discovered a new skill! I could say working on APPROACH increased my self-confidence.’ (PC member, UK)*

In addition, being part of a PC means being part of a group of peers that understand and support each other.

*‘I love to be able to think of other PC members as my friends as well as people I work with. Even “little” things like Jon sending us a beautiful photo of the Norwegian Forest make a difference.’ (PC member, UK)*

Overall, as the experience in APPROACH demonstrates, patient involvement may not always be easy and will need time to grow. But it is an enriching experience for patients and researchers involved, and has broad benefits that range beyond the scope of the research project.

*‘In large complex clinical studies, in which multiple partner organizations are involved, we tend to forget that our joint effort is not just scientific research or getting a new drug on the market, but it is finding actual medical solutions ‘for patients with patients’. The work of the PC constantly reminds us of this goal.’ (Programme Manager, The Netherlands)*

## Supplementary Information

**Additional file 1.** GRIPP-2 short form.

**Additional file 2.** Terms of Reference.

**Additional file 3.** Participant Questionnaire.

**Additional file 4.** Example participant newsletter.

**Additional file 5.** Description of ‘focus group’ activity with consortium at Annual Meeting 2019.

## Data Availability

Resources referred to in the text such as Terms of Reference and participant questionnaire template are appended to this article as supplementary materials. Other data such as notes taken during study participant visits and participant questionnaire results are not publicly available due to potential compromise of individual privacy.

## Abbreviations

DMOAD: Disease Modifying OsteoArthritis Drug
EC: European Commission
EFPIA: European Federation of Pharmaceutical Industries and Associations
EMA: European Medicines Agency
EULAR: European League Against Rheumatism
EU: European Union
FDA: Food and Drug Administration of the United States of America
IMI: Innovative Medicines Initiative
OA: Osteoarthritis
OARSI: Osteoarthritis Research Society International
PARE: People with Arthritis and Rheumatism
PC: Patient Council
PI: Principal Investigator

## Footnotes

1 https://www.imi.europa.eu/projects-results/project-factsheets/approach

2 Quotes were collected from researchers and PC members both during the 2019 Annual Meeting (see Suppl. File 5) and specifically in the process of writing this article. Quotes were selected for inclusion in this article based on their fit with the content presented.
