# Correction to: Making the patient voice heard in a research consortium: experiences from an EU project (IMI-APPROACH)

**DOI:** 10.1186/s40900-022-00348-8

**Published:** 2022-04-19

**Authors:** Jane Taylor, Sjouke Dekker, Diny Jurg, Jon Skandsen, Maureen Grossman, Anne-Karien Marijnissen, Christoph Ladel, Ali Mobasheri, Jon Larkin, Harrie Weinans, Irene Kanter-Schlifke, the APPROACH research consortium and APPROACH Principal Investigators

**Affiliations:** 1 The APPROACH Patient Council, Utrecht, The Netherlands; 2 Rheumatology and Clinical Immunology, University Medical Center Utrecht, Heidelberglaan 100, 3584 CX Utrecht, The Netherlands; 3 BioBone B.V, Amsterdam, The Netherlands; 4 University of Oulu, Oulu Finland State Research Institute Centre for Innovative Medicine, Vilnius, Lithuania; 5 University Medical Center Utrecht, Utrecht, The Netherlands; 6 GlaxoSmithKline, Collegeville, PA USA; 7 Lygature, Jaarbeursplein 6, 3521 AL Utrecht, The Netherlands

## Correction to: ***Res Involv Engagem*** 7, 24 (2021). 10.1186/s40900-021-00267-0

Following publication of the original article [1], the authors reported errors in the Acknowledgements section. The revised Acknowledgements section is indicated hereafter and the changes have been highlighted in **bold typeface**.

The incorrect Acknowledgements section reads:

The authors would like to thank all partners of the APPROACH consortium for their continued collaboration and support of the PC. Versus Arthritis (UK) and ReumaNederland (The Netherlands) have made a particular contribution at the project proposal stage and invited founding PC members. Ingrid Lether participated in the MRI test and re-test study (Dry run of clinical protocol by APPROACH researchers section), together with two co-authors of this paper. Merck KGaA kindly allowed the authors to utilize drawings from its 350th anniversary research day (Fig. 3); the artist was Andreas Gaertner from die-zeichner.de. The authors would also like to acknowledge two former PC members from Germany and Spain, respectively; when still active members of the PC, their contribution and experience was highly valued. The authors would like to thank specifically the IMI reviewers and expert panels who encouraged the involvement of patients from the inception of this project.

The correct Acknowledgements section should read:

The authors would like to thank all partners of the APPROACH consortium for their continued collaboration and support of the PC. Versus Arthritis (UK) and ReumaNederland (The Netherlands) have made a particular contribution at the project proposal stage and invited founding PC members. **Leonie Hussaarts (Lygature) was the PC coordinator from the start of the APPROACH project; in this role she initiated and managed a number of trivial PC activities (see section “Operationalizing the PC”).** Ingrid Lether participated in the MRI test and re-test study (Dry run of clinical protocol by APPROACH researchers section), together with two co-authors of this paper. Merck KGaA kindly allowed the authors to utilize drawings from its 350th anniversary research day (Fig. **5**); the artist was Andreas Gaertner from die-zeichner.de. The authors would also like to acknowledge two former PC members from Germany and Spain, respectively; when still active members of the PC, their contribution and experience was highly valued. The authors would like to thank specifically the IMI reviewers and expert panels who encouraged the involvement of patients from the inception of this project.

All the changes requested are implemented in this correction and the original article [1] has been corrected.
