# Supplementary material for: Making the patient voice heard in a research consortium: experiences from an EU project (IMI-APPROACH)
Source: Res Involv Engagem. 2021 May 10;7:24. doi: 10.1186/s40900-021-00267-0 (PMC8107424; doi:10.1186/s40900-021-00267-0)
Supplement: Supplementary file 1 — Additional file 1. GRIPP-2 short form. [file 40900_2021_267_MOESM1_ESM.pdf]

**Section and topic**	**Item**	**Reported on page**
1: Aim	Report the aim of PPI in the study	6
2: Methods	Provide a clear description of the methods used for PPI in the study	9-12
3: Study results	Outcomes—Report the results of PPI in the study, including both positive and negative outcomes	13-21
4: Discussion and conclusions	Outcomes—Comment on the extent to which PPI influenced the study overall. Describe positive and negative effects	21-34
5: Reflections/critical perspective	Comment critically on the study, reflecting on the things that went well and those that did not, so others can learn from this experience	21 - 39

GRIPP2 Short Form .
