# Supplementary material for: Making the patient voice heard in a research consortium: experiences from an EU project (IMI-APPROACH)
Source: Res Involv Engagem. 2021 May 10;7:24. doi: 10.1186/s40900-021-00267-0 (PMC8107424; doi:10.1186/s40900-021-00267-0)
Supplement: Supplementary file 2 — Additional file 2. Terms of Reference. [file 40900_2021_267_MOESM2_ESM.pdf]

Terms of Reference APPROACH Patient Council | 31 May 2017

Introduction APPROACH

The IMI-funded APPROACH* project was established in 2015 and aims to develop a set of guidelines that allow for the selection of patients to support clinical trials, with medication that is specifically designed for subsets of OA patients. To this end, the consortium will set up a biomedical database of different OA patients, as well as a longitudinal cohort to validate and qualify methods for patient stratification. In total, 25 partners throughout Europe and one partner from the United States are involved. The project runs from June 2015 – December 2020 and is led by Jonathan Larkin (GlaxoSmithKline) and Harrie Weinans (UMC Utrecht).
** APPROACH: Applied Public-Private Research enabling OsteoArthritis Clinical Headway*

Tasks of the Patient Council

The Patient Council consists of 5 – 7 patients and was established to advice the APPROACH project on the patient perspective. Specifically, the council will:

- Provide advice regarding the clinical trial design in WP3 - **2015 & 2016**
- Participate in editing the (translated) informed consent forms in WP3 - **Jan 2017 to July 2017**
- Help write communication materials for patients in the clinical trial, for example: flyer, newsletter - **2017 onwards, until the end of the trial**
  The trial will start after summer and continue for approximately 2.5 years. WP3 would like to send a newsletter but the details are not clear yet.
- Attend the Annual Meeting to follow progress and (optional) to give a presentation - **once a year in October / November until the projects ends in 2020)**

To make the Council more autonomous, a Chair will be appointed, who will discuss with the Patient Council (via e-mail or WhatsApp) the preferred approach for completing the tasks. If support is required, the Chair will contact Lygature.

Tasks of APPROACH Work Package 4 Leadership

- Inform / update the Patient Council on a regular basis by means of monthly updates (e-mail) and teleconferences scheduled as needed, but at least once every three months
- To communicate timelines well in advance
- To ensure that the Council has at least 20 days for review of documents
- To facilitate communication between the members of the Council, for instance through MyProjectPlaza or organizing teleconferences
- Lygature specifically: To act as the secretary and primary point of contact for the Council

Tasks of Charities: Reumafonds/DAF and Arthritis Research UK

- To provide independent advice about involving patients in research when needed.

Annual Meetings

Because of the scientific nature of the project, the Annual Meetings are very much content-driven and technical, which might make it difficult to follow the details. Nevertheless, the APPROACH project would highly appreciate the presence of the Patient Council for three reasons:

1) To keep the Council informed about progress
2) To give the Council an opportunity to get to know each other
   From now on, there will be time for this in the schedule
3) To keep the researchers focussed on the goal: improving treatments for (future) patients

The Council will be invited to give a presentation during the Annual Meeting. The Council is free to choose the topic, together with or with help from Work Package 4 Leadership.

Remuneration

All expenses made while traveling for the project can be declared using the reimbursement form from the Utrecht University Medical Center. Help with reimbursement is provided by [NAME ADMIN SUPPORT] from the UMCU.

From 2017 onwards, we would like to provide the Council with an allowance for their work.

- Members of the Patient Council who actively participate in the discussions throughout the year will receive remuneration of 150 euros on a yearly basis.
- Members of the Patient Council who attend the Annual Meeting will receive additional remuneration of 150 euros per Annual Meeting.

Disclaimer

This is not a legal document and the contents within it are not binding.

- *[NAME PC COORDINATOR], 31 May 2017*
