# Supplementary material for: Making the patient voice heard in a research consortium: experiences from an EU project (IMI-APPROACH)
Source: Res Involv Engagem. 2021 May 10;7:24. doi: 10.1186/s40900-021-00267-0 (PMC8107424; doi:10.1186/s40900-021-00267-0)
Supplement: Supplementary file 3 — Additional file 3. Participant Questionnaire. [file 40900_2021_267_MOESM3_ESM.pdf]

EVALUATION FORM APPROACH visit

		YES	NO	Not applicable
1	Were you given enough notice for your APPROACH visit?			
2	Was it clear to you at what time and where you were expected to register for the APPROACH visit?			
3	Did you know what to expect (per item) during the APPROACH visit (physical examination, radiographs etc.)?			
4	Could you easily find the route to the different departments (radiology, rheumatology)?			
5	Were all departments easily accessible?			
6	Did you feel it was difficult / aggravating to complete the pain diary?			
7	Did you find the questionnaires useful?			
8	Is the catering (food and drinks) good and sufficient ?			
9	Is the research visit at the end of the day aggravating ?			
10	Was there any more information that you would have liked included in the information letter beforehand?<br><br>If yes, please specify			
11	Was there any more information you would have liked to have during the APPROACH visit?<br><br>If yes, please specify			
		**GOOD**	**BAD**	**Not applicable**
12	How did you experience the treatment by the employees of the Radiology department ?			
13	How did you experience the treatment by the research physicians at the Rheumatology department?			

How could we make the APPROACH visit more comfortable for you ?

....

....

Is there any other information you would like to add to this form? Yes/No. If yes, please specify.

....

....

You could also send an e-mail to the patient council of APPROACH: patientcouncil@lygature.org
