# Supplementary material for: Making the patient voice heard in a research consortium: experiences from an EU project (IMI-APPROACH)
Source: Res Involv Engagem. 2021 May 10;7:24. doi: 10.1186/s40900-021-00267-0 (PMC8107424; doi:10.1186/s40900-021-00267-0)
Supplement: Supplementary file 4 — Additional file 4. Example participant newsletter. [file 40900_2021_267_MOESM4_ESM.pdf]

You are receiving this newsletter because you are taking part in the APPROACH project, as a patient, researcher or doctor. This newsletter will keep you informed on project progress roughly once every six months. Please also visit our website: www.approachproject.eu.

approach

NEWSLETTER

NUMBER 5

Applied Public-Private Research enabling OsteoArthritis Clinical Headway

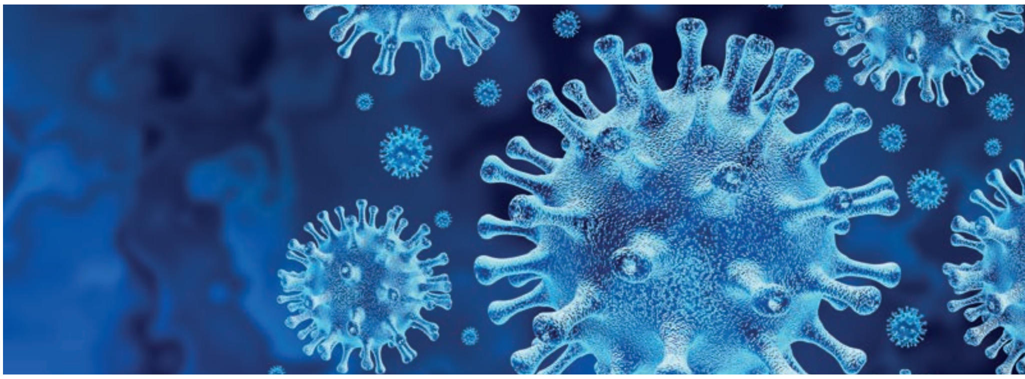

WELCOME

to the fifth edition of the APPROACH newsletter!

In this edition, we will focus on the consequences of the COVID-19 pandemic for APPROACH, the contribution of APPROACH for the development of drugs for osteoarthritis, the analysis done in laboratories and the use of machine learning and data bases in the project. As usual, we will also give you an update of the Patient Council activities for the past six months. We hope you enjoy reading our update!

Progress and COVID-19 consequences for APPROACH

In March, an important new step was reached for the APPROACH study: around 20 patients performed their last M24 visit and finished the study. For these patients, experts performed a longitudinal evaluation of osteoarthritis shown at MRIs taken at baseline, 6, 12, and 24 months

After this good news, the COVID-19 crisis occurred and unfortunately the clinical sites were not able to perform new M24 visits and remaining M12 visits.

In regular care, almost all patient visits in the hospitals were replaced by phone calls, to prevent spreading of the virus as much as possible. Similarly, also your visits to the clinical sites for the APPROACH study were cancelled.

After two months, in mid-May, it was nice to hear that clinical sites in Norway, Spain, and France are able to restart APPROACH visits within a few weeks. Presently, the sites in The Netherlands are not allowed yet to perform APPROACH visits, but hope to follow soon. This is good news for the APPROACH study, because in this way the impact on the APPROACH study will be limited and the quality of the study will not be jeopardized by the crisis.

Before the re-start of the visits, the clinical sites confirmed that they will perform the upcoming visits according to all regulations including COVID-19-related regulations prescribed by their local authorities. The clinical sites were asked to share their experiences with these planned patient visits during COVID-19 restrictions in order to have the best conditions for a safe APPROACH visit.

Additionally, in cooperation with the patient council, we gave some guidelines to the clinical sites to preserve safety of the participants and personnel:

1. take into account that the APPROACH population may be at high risk
2. keep physical distance as much as possible and stick to hygiene measures
3. keep in mind/ organize that patients can travel to the hospital in a safe way
4. do not plan too many patients on one day, in order to give them the opportunity to keep distance
5. clean materials, such as GaitSmart, HandScan, etc. after each usage.

The APPROACH clinical sites do everything they can to decrease infection risk during the visits. We ask you also to respect the prescribed COVID-19 measures during your everyday life in order to stay healthy and safe.

Authors: Agnes Lalande, Floris Lafeber, Anne Karien Marijnissen, Work Package 3 – Cohort and Imaging

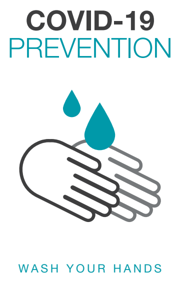

Drugs against osteoarthritis

Today, for OA only drugs against pain, not against structure damage, are on the market. Two pharmaceutical companies involved in APPROACH, Servier and Merck, are working on drugs against structure damage in osteoarthritis. Servier is working on a drug against an enzyme involved in cartilage degradation (phase II). Merck, is developing another drug against the same enzyme (phase I). In addition, two other products against pain in OA are in development: one intra-articular product (phase III) and one product in the last phase before approval for use in clinical practice.

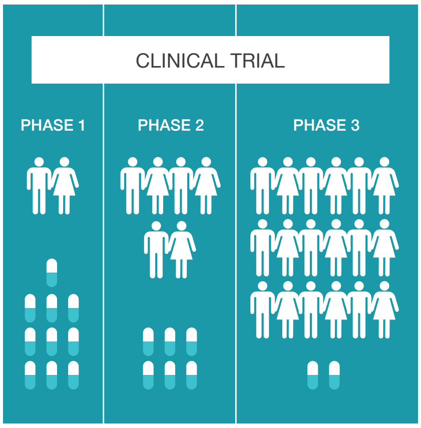

From small numbers of healthy volunteers to large numbers of patients.

To improve drug development for osteoarthritis, a clear definition of different OA types is important. The APPROACH study – thanks to you as a participant - will contribute to this definition and will allow the development of guidelines for differentially diagnosing the right patient for the right treatment.

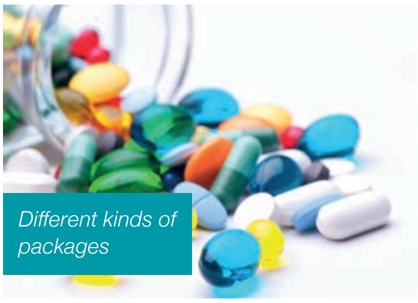

Different kinds of packages

Authors: Agnes Lalande, Floris Lafeber, Anne Karien Marijnissen, Work Package 3 – Cohort and Imaging

What happens to real data and what will the results from the study mean?

All data collected during the visits are processed and stored in two places. The clinical data are entered into a data warehouse running on the tranSMART platform, which is a free software developed collaboratively by researchers and pharmaceutical companies. Raw images, for example radiographs or MRI scans, are stored separately using another free software platform called XNAT. Members of the APPROACH project will be able to access the data in tranSMART and analyze them. Within the project we have several different groups looking at different subsets of the data. For example, four different teams are analyzing the knee X-rays, three are looking at MRI scans, another three work with CT scans. We also have teams working with hand and hip images, a team analyzing the motion sensor data from the GaitSmart devices, and several teams analyzing the blood, serum and urine samples. The results of this work will be shared between members through tranSMART.

Most of the data will be used to test a research hypothesis about the OA disease. For example, a question about whether a risk of developing the disease is higher in one group of patients than the other, or whether some biomarker can indicate a future disease progression. At some point these data will become available to scientists outside the APPROACH consortium, so that they could analyse the data and find answers to their own research questions. The main research objectives of the project are, however, focused around discovery of OA subtypes. If identified, this subtypes might make the drug development easier, as a specialized treatment might be less complicated, than one that has to work for all patients. We are currently using methods from the field of machine learning to automatically search for patterns in the data and identify such subtypes. For now, we focus on the data from the first two visits, but in the future we will mainly look at the differences between the beginning and the end of the study.

Author: Pawel Widera, Work Package 1 - Bioinformatics

Quick Facts

‘ I STOPPED USING THE WORD SUBJECT AND USED PATIENT INSTEAD.’

The Patient Council (PC) asked the researchers at the annual meeting of Approach in 2019 to write down their individual opinion about having a PC as part of the project. Here is a summary of their answers:

1. The PC made the patient visible.
2. The PC has an evidently positive effect on patient enrolment, retention/adherence and logistics in clinical trials.
3. The PC provided an invaluable insight into what patients find specifically important.
4. The PC provided ‘ tremendous’ motivation to scientists/physicians involved.
5. Members of the PC are asking surprisingly insightful questions that lead to new directions for research.
6. Changes as a result of the input of the PC: better communication; interaction with patients; better perception and understanding; improved logistics.
7. The PC can provide insight in the next stages of APPROACH through more involvement of patients in future studies.

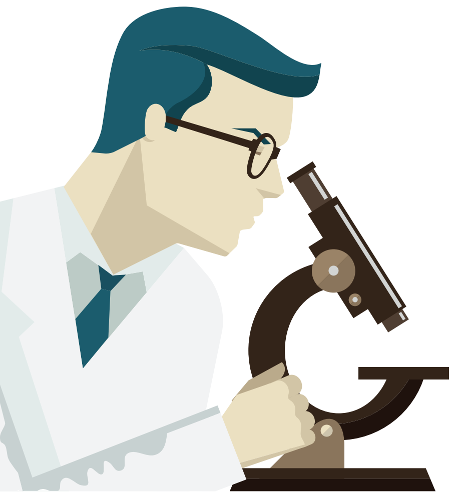

APPROACH contributes to the development of drugs for osteoarthritis

The pharmaceutical development of a new drug is a complex process, which takes several years. The process is divided into different phases: screening, pre-clinical development, clinical trials, and finally registration by national authorities in different countries.

Actually, most compounds fail before the registration phase, which is also the case for most drugs against osteoarthritis (OA). Many companies have even abandoned OA drug development. One of the problems is the lack of measures to identify patient benefit from a therapy at the level of pain and tissue modification. Another issue might be that the general OA population is very diverse. Not all osteoarthritis patients have similar disease characteristics. Some have more cartilage related problems for example, while others have more bone or inflammation related complains.

Collaboration between universities and pharmaceutical companies

Within the APPROACH project universities and pharmaceutical companies are working together to identify different types of knee OA, which will facilitate the process of drug development in the treatment of osteoarthritis. The set-up of the APPROACH cohort is based on innovative methods that identifies different types of knee OA. One of the objectives is to define subgroups of progression of knee OA, with similar disease characteristics, for example bone derived complains, or a diminished specific cartilage compound, or a specific kind of pain.
Knee OA progression in the APPROACH participants is evaluated in detail based on structural damage and on symptoms. Structural damage is measured by different imaging techniques such as MRI, CT and radiography. Additionally, changes in tissues are measured by biomarkers in blood and urine. If cartilage of the knee has been damaged for example, components of the cartilage can be found in blood and urine. Symptoms of knee OA are measured by several questionnaires and functional tests, all measuring a specific part of the symptoms. For example, we measure different kinds of pain and different kinds of diminished functional abilities.
Identification of subgroups would enable us to develop treatments or pharmaceutical compounds directed to specific disease characteristics, for example a compound specifically directed to bone changes. Patients belonging to this subgroup will have a bigger chance to benefit from such a treatment compared to patients belonging to another subgroup.

Drug development

The first step in the pharmaceutical development of a new drug is **the screening phase**. A new drug is developed against a specific cellular or molecular structure involved in the pathology. A huge amount of compounds is tested for their ability to modify the specific cellular or molecular structure involved in the disease. Then, the activity of the most selective compounds is being tested in a disease model (cells or animal models) to see if the disease can be reversed.

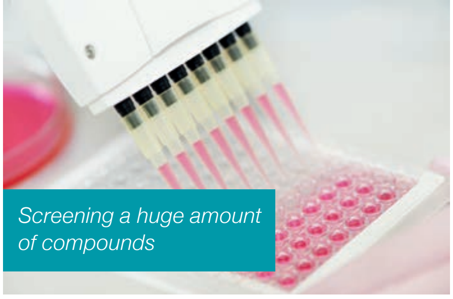

Screening a huge amount of compounds

Once a lead compound series has been established with sufficient potency and selectivity for the specific molecular or cellular structure involved in the pathology, one or two compounds will be tested in **the Pre-clinical Development phase**. Since after the screening phase little is known about the compounds' safety, toxicity and behavior in the body, these items will be assessed in animals prior to human clinical trials, i.e. during the Pre-Clinical Development phase. Also chemical makeup, stability, and solubility of these compounds are established. In addition, the production process is scaled up from milligram scale to kilogram and ton scale. Concurrently, the suitability for package as capsules, tablets, aerosol, intramuscular or subcutaneous injectable, or intravenous formulations is studied.

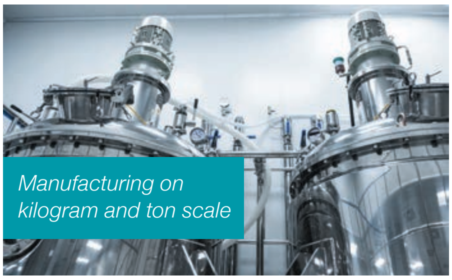

Manufacturing on kilogram and ton scale

The last step before registration of a drug concerns **Clinical trials**, subdivided in three phases:

- Phase I, in healthy volunteers, to determine safety and dosing.
- Phase II, in small numbers of patients having the disease targeted by the new drug to get information on efficacy and safety
- Phase III, in large numbers of patients having the disease targeted by the new drug to determine safety and efficacy

In parallel to clinical trials, long-term or chronic toxicities in animals (on fertility, reproduction, immune system, cancer) are defined. If a compound has an acceptable toxicity and safety profile, and the desired efficacy, the compound is submitted for marketing approval in various countries. After approval by national authorities, the drug will be registered and can be prescribed by the doctor.

What is analyzed in participant samples, and why is that important?

The blood samples, collected during the first clinical visit, have been distributed to the research laboratories where they have been analyzed. For the analysis, 3 different techniques were used:
1) with "lipidomics" the lipid (important components of living cells) composition of the blood could be determined,
2) with "transcriptomics" we investigated ongoing changes (up or down) in biological processes in the body which are reflected in the blood, and
3) with "biochemical markers analysis", which is the measurement of several specific biochemical substances, the current status of the disease could be determined. An additional technique that will also be performed soon is "proteomics" (set of proteins) that measures the proteins composition in the blood. The laboratory work of the first 3 techniques has already been completed and these data will now be used for "data mining", a computer technology that can discover patterns in large datasets.

Combining the information from these technologies will help us to link different processes that are important in the progression and outcome of OA. The first results are expected early 2021.

With the results we will be able to identify the most important destructive biological processes for which ultimately new drugs can be developed.

Author: Anne-Christine Bay-Jansen, Work Package 2 - Biomarkers

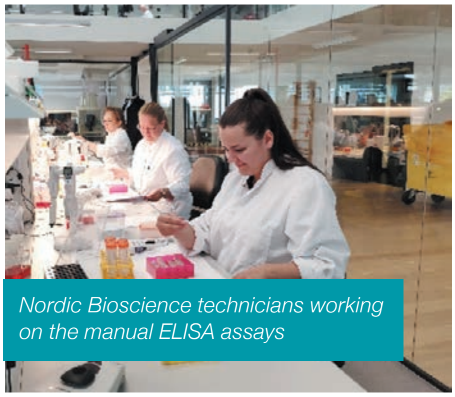

Nordic Bioscience technicians working on the manual ELISA assays

Activities of Patient Council members

APPROACH Patient Council member **Jon Skandsen** held several presentations in Oslo to advocate for Patient Engagement (PE) in research in general, but also to tell about the experiences drawn from the work in the APPROACH project. The first presentation took place in January at one of the research departments at the Diakonhjemmet Hospital. The audience was composed of doctors, researchers and scholarship holders. A week later the same presentation was held for a forum of PE representatives, i.e. patients. An important topic was to explain how the PE work is implemented in APPROACH. Furthermore, in February Jon participated with a presentation on a strategy seminar for the new research division at the Diakonhjemmet Hospital. There were approximately 50 doctors and researchers present and a lot of positive feedback was received on the PE presentation. The APPROACH Newsletter was also handed out as an example on patient involvement and communication. Finally, in late February, Jon presented at the Oslo Metropolitan University, Faculty of Health Science. The presentation was given to professors, researchers and PhD students where Jon elaborated on the European League Against Rheumatism (EULAR) Guidelines for patient involvement in research projects. Jon explains: "The audience was quite interested and also impressed by the APPROACH project. They understood the challenges of patient involvement in such a large, multi-national project, and also realized that a lot of efforts are required for such a project to be successful."

Maureen Grossman, a member of the PC shares: "When I was diagnosed with osteoarthritis I volunteered to join the charity Versus Arthritis as a "patient expert". This led to my representing the charity when it became a partner in APPROACH, I became a member of the Patient Council. Working with colleagues in APPROACH became a significant part of my life. I was proud of the team. I felt I should let the world know! I did not manage the world, but I am a member of the University of the Third Age and I have been giving lectures to Science Groups. I use the title "Arthritis - where are we going?" This gives me the opportunity to discuss APPROACH, explaining about patient involvement and the importance of collaboration in research. Many attending have osteoarthritis and ask pertinent questions about patient benefit and science. I finish by distributing our newsletters and brochures to make sure APPROACH is valued and remembered."

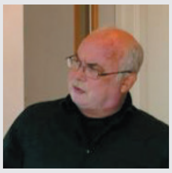

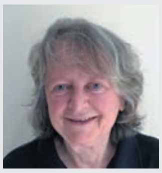

APPROACH: Applied Public-Private Research enabling OsteoArthritis Clinical Headway
The research leading to these results has received support from the Innovative Medicines Initiative Joint Undertaking under grant Agreement n° 115770, resources of which are composed of financial contribution from the European Union's Seventh Framework Programme (FP7/2007-2013) and EFPIA companies' in kind contribution. See www.imi.europa.eu.
