# Supplementary material for: Making the patient voice heard in a research consortium: experiences from an EU project (IMI-APPROACH)
Source: Res Involv Engagem. 2021 May 10;7:24. doi: 10.1186/s40900-021-00267-0 (PMC8107424; doi:10.1186/s40900-021-00267-0)
Supplement: Supplementary file 5 — Additional file 5. Description of ‘focus group’ activity with consortium at Annual Meeting 2019. [file 40900_2021_267_MOESM5_ESM.pdf]

This document describes the session at the 2019 APPROACH annual consortium meeting, where the Patient Council (PC) collected feedback from researchers on the work of the PC thus far and ideas for future contribution.

About the session:
Jane Taylor chaired the 1 hour plenary session. Other PC members joined her on stage and helped with handing out feedback cards. Part 1 of the session focused on 'Looking back: PC activities of the past year'. Part 2 was aimed to trigger ideas for the future: 'Researcher feedback & Open discussion'. Slides were presented for part 1, reminding the audience of the role of the PC and describing its activities of the past year.

Methodology to collect feedback:
For part 2, the following question were put up on a slide:
Question 1: What have been the positives for you of having a Patient Council as part of the project?
Question 2: What have you changed or done differently (if anything) as a result of their input so far?
Question 3: How do you think the Patient Council could most effectively provide insight in the next stages of the APPROACH project?

The audience was given 10 minutes to reflect on these questions and anonymously write answers on feedback cards handed out. Cards were received by 45 researchers. The PC then sorted the cards and distilled common topics summarized below.

Feedback collected:

<u>Feedback on Question 1</u>
1. The PC has had a positive effect on participant enrolment, retention / adherence and logistics in clinical trials through input to the protocol and participant communications (e.g. newsletters). To hear from patients whether what we planned was doable changed in a positive way the researchers' thinking on what participants were prepared to do in tests.

2. The PC provided insight into what patients find specifically important to help direct communications to participants and anticipate questions they might ask in the course of the trial.

3. PC provided 'tremendous' motivation to scientists/physicians involved. Reminding scientists why we are doing this project, what the long term goals are, keeping scientists focused on what is important, keeping things realistic – 'why are we measuring this? Why are we doing things in this way?'. Forcing scientists to think out of their 'standard' box regarding outcomes. Giving researchers a sense that they are part of a bigger community fighting OA.

4. The PC asked surprising questions that lead to new directions for research. Asking practical ''down to earth'' questions has helped researchers see another (patient) perspective.

5. The PC makes the patient reality visible. PC members represent different problems/stages of OA and provide researchers with a better understanding of disease impact. This helps change perceptions and makes researchers realise that having OA doesn't 'stop you from being a happy and fully engaged individual.'
''When you work with data it is very easy to forget what the numbers actually *mean.''*

<u>Feedback on Question 2</u>

1. Changing communications. The connection with the PC has made researchers rethink how they communicate their work. It triggered discussion if and how individual information/data gathered from APPROACH could be shared with participants.
'' I stopped using the word *'subject'* and used *'participant'* instead. Originally I was told off, subject is not necessarily a patient yet, only a candidate so in a strict meaning more correct. But it sounds *awful….''*

''I presented my work differently, with a more layered approach so that the key intuition behind the concept is set out first and detail *later..''*

2. Interactions with patients.
'Interact directly with patients, something not done *before.''* Understand limitations and ''humanise'' biomedical research; understand what 'success' is for patients (which may not be the same as for researchers). As a whole group we have tried to incorporate and improve medical test conditions. Learned to involve patients more actively.

3. Changing perceptions and understanding.
The PC helped researchers increase their understanding of how the disease affects people. It also helped researchers better understand some things that need to be considered for trial design: e.g. better balancing the numbers of trial participants receiving placebo vs treatment and better taking into account the final goal of research (for patients).
'*'With* new members of the lab, I now discuss not only the science of OA but also how it affects the *patient.''*

4. **Practical changes.**
For example the patient invitation letter was changed to take into account timings in relation to travel distance and logistics of patient visits were adapted.

''This test should be *repeated''* is something I rarely say anymore. The inconvenience for the patient is something I tend to keep in mind *now.''*

Feedback on Question 3

1. Communication of results. Help researchers think about how to communicate results of research in a way that is also accessible to patients. What final results should be communicated back to study participants and how? Think about what the PC would like to see evaluated from the cohort data.

2. Help in writing articles. PC could help researchers improve the readability of scientific articles, ask more ''so what'' questions about the outcomes of APPROACH so that we keep focusing on things that matter to patients rather than abstract goals.

3. Dissemination. Continue to disseminate the project work to a wider audience. Explain to politicians and patients why work like this is critical for the future of personalised healthcare. The PC has an Ambassador role for the project to patients and others in OA community, at European and individual country levels.

4. More involvement in future studies to reduce the burden of clinical study protocols for participants and think ahead to next stages. Help to think about future use of study results. ''If we want to validate the outcomes for a clinical tool, then we need advice on how to design a new study and how this tool can help doctors and patients in the future. This will help in shaping something that is actually effective in the *clinic*'' Paper publication on improving design of future trials. Advice on what should be communicated to patients in the future if they enter a trial.

5. PC should do 'more of same'. Continue to interact with participants through e.g. newsletters. Keep asking critical questions from patient point of view. Contribute to creating a template of letters to study participants. Design another evaluation form for participants to get learnings for future studies. Design a communication channel which allows for feedback between patients and clinician for test results.

'I think that the PC is doing an awesome task and they must continue this way'
